# Research on the Sensing Characteristics of an Integrated Grid-like Sensor Based on a Triboelectric Nanogenerator

**DOI:** 10.3390/s24030869

**Published:** 2024-01-29

**Authors:** Shiyu Zhao, Guanghui Han, Huaxia Deng, Mengchao Ma, Xiang Zhong

**Affiliations:** 1 School of Instrument Science and Opto-Electronics Engineering, Hefei University of Technology, Hefei 230009, China; syzhao@mail.hfut.edu.cn (S.Z.);; 2 CAS Key Laboratory of Mechanical Behavior and Design of Materials, Department of Modern Mechanics, University of Science and Technology of China, Hefei 230027, China

**Keywords:** triboelectric nanogenerator, self-sensing, velocity sensing, displacement sensing, integration

## Abstract

With the development of the integration and miniaturization of sensing devices, the concept of self-sensing devices has been proposed. A motion state is self-sensed via the structure or integration of an actuator in the construction of a sensing unit. This device is then used to capture the perception and measurement of states such as position, displacement, and speed. A triboelectric nanogenerator converts mechanical energy into electrical energy through the coupling effect of contact generation and electrostatic induction, which represents one of the reliable ways through which to realize integrated sensing. In this world, the power generation technology of the TENG is applied to a sensing device. The sensing characteristics of a grid-like TENG are designed and analyzed in freestanding triboelectric mode. Firstly, a relation model of displacement, velocity, voltage, and charge is established. The charge-transfer increment and current amounts are linearly related to the velocity. The open-circuit voltage has a positive relationship with the displacement. The maximum open-circuit voltage and the maximum charge transfer are fixed values, and they are only related to the inherent parameters of a triboelectric nanogenerator. Next, the sensor model is constructed using COMSOL Multiphysics 6.0. The simulation results show that the relationships between output voltage and charge transfer, as well as those between the increments of charge transfer, velocity, and displacement, are consistent with the results derived from the formula. Finally, a performance test of the designed sensor is carried out, and the results are consistent with the theoretical deduction and simulation. After analysis and processing of the output electrical signal by the host computer, it can feedback the frequency and speed value of the measured object. In addition, the output signal is stable, and there is no large fluctuation or attenuation during the 521-s vibration test. Because the working unit of the sensor is thin filmed, it is small in size, easy to integrate, and has no external power supply; moreover, it can be integrated into a device to realize the self-sensing of a motion state.

## 1. Introduction

State monitoring primarily involves the assessment of factors such as velocity, acceleration, and vibration frequency. In general, the motion states of mechanisms are monitored using contact measurement [1,2] via external sensors or visual measurement [3,4,5]. Piezoelectric and capacitive sensors are the most prevalent types used for acceleration measurement. Their utilization necessitates careful consideration of the sensor installation space and the potential impact of sensor quality on the system dynamics [6,7]. Utilizing visual methods requires consideration of high camera costs, environmental interference, and the complexity of the measurement method [8]. Therefore, researching a widely applicable, low-impact, and cost-effective sensing device that can provide real-time monitoring of the frequency, displacement, or velocity of operating equipment holds significant importance.

Integrating a sensing device allows the actuator to self-sense its motion, eliminating the need for external sensors and minimizing additional device introduction [9,10,11]. Research into the self-sensing functionality of actuator displacement and velocity has made some progress—for instance, by integrating electromagnetic induction devices into moving mechanisms. This utilizes relative motion, causing coils to move in a magnetic field, generating electrical signals related to displacement or velocity [12,13,14,15]. Its sensing mechanism, based on Faraday’s law of electromagnetic induction, requires a stable and relatively high operating frequency (above 50 Hz), as its output power is proportional to the square of the frequency [16,17]. This makes it less suitable for low-frequency motion. Capacitive and piezoresistive sensors rely on external power sources, limiting their application in self-sensing functions [18,19].

Wang first proposed the concept of the triboelectric nanogenerator (TENG) in 2012 [20]. Its principle lies in the coupling of the triboelectric and electrostatic induction effects between materials of different electrode polarities upon contact, efficiently converting minute mechanical energy into electrical power [21]. Due to its simple structure [22,23,24], rich materials options [25], cost-effectiveness [26], and versatility in manufacturing, the TENG has been applied to harvest energy from diverse environments like vibrations, the tide, wind, and human movement [27]. The energy harvesting method of the TENG is in line with the concept of green energy promoted today. At the same time, it can also be used as a self-powered supply unit of sensing devices, providing energy support for wireless distributed sensing and smart wear devices [28,29]. The TENG operates in four main modes: contact separation mode, lateral sliding mode, single-electrode mode, and freestanding triboelectric layer mode [30]. By investigating the relationship between output electrical signals and motion states in different operating modes, one can establish parameter models related to speed, displacement, and frequency. Based on this foundation, researchers have designed corresponding sensing devices according to the requirements of specific application scenarios, where the output electrical signals can reflect the motion state of the measured object [31,32]. Since the TENG is a thin-film sensor, its small size and flexibility make it easy to integrate into various motion devices. Therefore, sensors based on TENGs offer an effective means by which to achieve integration between actuators and sensors.

In this paper, the relationship between open-circuit voltage, short-circuit current, and motion speed is analyzed by deducing the formula for a TENG with a freestanding triboelectric layer mode. Based on the analysis results, a TENG-based sensor structure was designed. A simulation model was built to analyze the relationship between the output electrical signal and the motion state of the sensor. At the same time, the influence of the gaps in the grid structure on the measurement accuracy of the sensor was qualitatively analyzed via simulation. Finally, the sensor was tested to verify the feasibility of a grid-like triboelectric layer TENG as an integrated self-sensing device.

## 2. Principle

### 2.1. Structure and Working Principle

**Figure 1:**
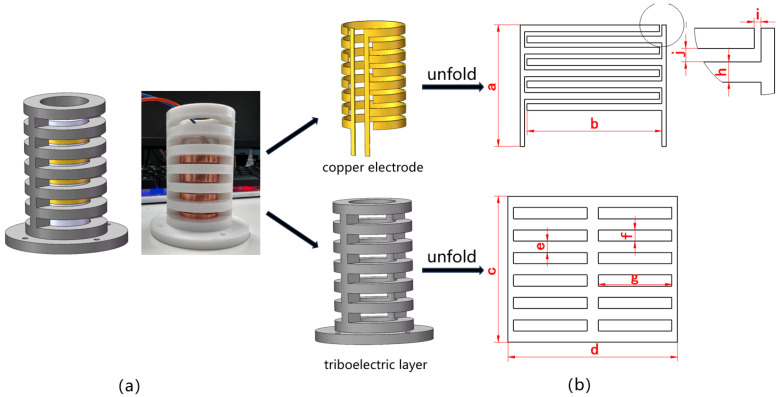
Schematic diagram of the integrated grid-like sensor: (**a**) structure and principle prototype; (**b**) structure parameters.

The designed sensing device is based on the freestanding triboelectric layer mode of a TENG. Its structure is shown in Figure 1a, which consists of a freestanding triboelectric layer with a grid structure on the outside and two groups of staggered metal electrodes in the same plane on the inside. According to the triboelectric sequence, polytetrafluoroethylene (PTFE) is selected as the material for the freestanding triboelectric layer, and copper is selected as the electrode material. The triboelectric layer is a grid cylinder, and its unfolded structure is shown in Figure 1b. The width of the individual grids and the gaps between the grids is 5 mm. The metal electrode is realized via the pasting of two interleaved copper foil gratings onto the surface of the acrylic cylinder. The width of the single-electrode grid is 3 mm; the width of the gaps between the grids belonging to different electrodes is 2 mm. The main structural parameters of the sensor are shown in Table 1.

**Figure 2:**
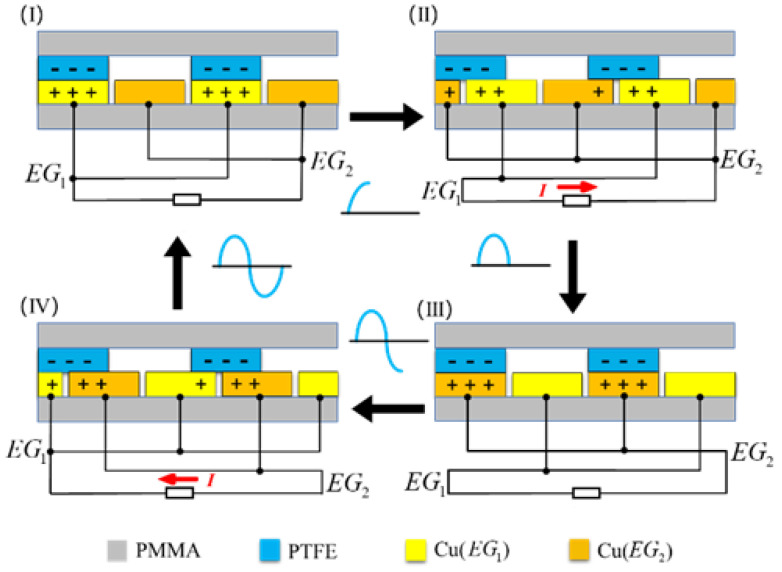
Schematic diagram of the charge transfer of the grating-structured freestanding triboelectric layer nanogenerator. (I): The PTFE layer overlaps with EG1. (II): The PTFE slides from EG1 to EG2. (III): The PTFE layer overlaps with EG2. (IV): The PTFE slides from EG2 to EG1.

In order to more intuitively describe the working principles of a grid-like TENG in freestanding triboelectric layer mode within a closed-loop circuit, a cross-section of the structure is taken to demonstrate the charge transfer during operation, as shown in Figure 2. The selected section includes two triboelectric PTFE layer grid bars, two copper electrode EG1 grid bars, two copper electrode EG2 grid bars, and an acrylic (PMMA) material base. The triboelectric PTFE layer is located at the top, and the copper electrodes—EG1 and EG2—are staggered at the bottom. The grid bars on the same piece of electrode are connected by a wire, and the two different electrodes are connected at both ends of the load in the external circuit.

During operation, because the electrical polarity of the triboelectric PTFE layer is opposite to that of the two materials of the copper electrode, the two materials generate contact charges on the surface following contact. The triboelectric PTFE layer is negatively charged, and the copper electrode is positively charged; it can be seen from the conservation of the charge that the amount of charge on the two materials is equal. The initial state is shown in Figure 2, where the triboelectric PTFE layer is completely coincident with electrode EG1 and is in electrostatic equilibrium, at which time there is no charge transfer between electrode EG1 and electrode EG2. When the electrode layer begins to slide to the right, the triboelectric PTFE layer gradually slides to the left, from an overlapping position with electrode EG1 toward electrode EG2. At this time, a potential difference between the two electrodes is also gradually generated, driving the negative charge from electrode EG2 to electrode EG1, and thus forming a current in the external circuit. The direction of this current is opposite to the direction of charge movement. As the electrode layer continues to move, the triboelectric PTFE layer overlaps with electrode EG2. At this point, the charge is no longer transferred, and another state of electrostatic equilibrium is achieved.

When the copper electrode then moves to the right, the triboelectric PTFE layer continues to slide to the left. Although the relative direction of motion is unchanged, the triboelectric layer slides from electrode EG2 to electrode EG1. The resulting potential difference causes the negative charge to be attracted and flow back to electrode EG2, creating a reverse current in the loop. When the triboelectric PTFE layer again overlaps with electrode EG1, the charge transfer ends. The whole process forms a periodic electrical signal in the external circuit.

### 2.2. The Relationship between Current and Speed

The electrical signals produced by the TENG result from the relative motion between the freestanding triboelectric PTFE layer and the metal copper electrode in the operational state, generating triboelectricity and electrostatic induction. Consequently, there is a certain correlation between the output electrical signal and the motion state, theoretically enabling the TENG to be employed to characterize the motion state.

**Figure 3:**
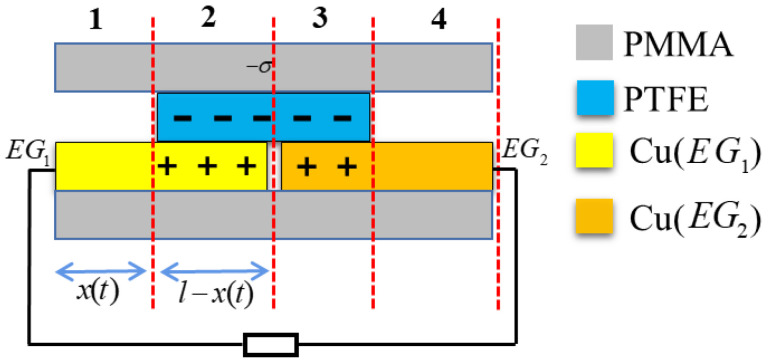
Charge transfer of a TENG within an external circuit. Region 1:the non-overlapping surface of electrode EG1. Region 2: the overlapping surface of electrode EG1. Region 3: the overlapping surface of electrode EG2. Region 4:the non-overlapped surface of electrode EG2.

First, consider the scenario of a closed-loop circuit with an external load, as illustrated in Figure 3. Let σ denote the friction charge density on the surface area ds of the independent triboelectric PTFE layer. According to the law of conservation of charge, the total charge on the corresponding copper electrodes EG1 and EG2 is σbds. When there is relative sliding between the triboelectric PTFE layer and the metal copper electrode, the charges on electrodes EG1 and EG2 can be expressed as follows:

(1) $$\frac{dQ_{1}}{C_{1}\left(s\right)}=\frac{dQ_{2}}{C_{2}\left(s\right)}$$

(2) $$dQ_{1}+dQ_{2}=\sigma bds,$$

where $dQ_{1}$ is the amount of charge in the ds region of electrode EG1, $dQ_{2}$ is the amount of charge in the ds region of electrode EG2, and $C_{1}\left(s\right)$ is the capacitance between the ds region on the surface of the triboelectric PTFE layer and electrode EG1. $C_{2}\left(s\right)$ is the capacitance between the ds region on the surface of the triboelectric PTFE layer and electrode EG2, and *b* is the width of electrodes EG1 and EG2. The following two formulas can be obtained by combining Formulas (1) and (2).

(3) $$dQ_{1}=\frac{\sigma bds}{1+\frac{C_{2}\left(z\right)}{C_{1}\left(z\right)}}$$

(4) $$dQ_{2}=\frac{\sigma bds}{1+\frac{C_{1}\left(z\right)}{C_{2}\left(z\right)}}.$$

The total charge on electrodes EG1 and EG2 is the integral of each small triboelectric region ds. Therefore, the total charge on electrodes EG1 and EG2 can be derived by integrating Formulas (3) and (4):

(5) $$Q_{1}=\sigma b\int_{0}^{l}\frac{ds}{1+\frac{C_{2}\left(s\right)}{C_{1}\left(s\right)}}$$

(6) $$Q_{2}=\sigma b\int_{0}^{l}\frac{ds}{1+\frac{C_{1}\left(s\right)}{C_{2}\left(s\right)}},$$

where *l* is the width of the metal copper electrode and the triboelectric PTFE layer.

During the relative motion between the triboelectric PTFE layer and the metal copper electrodes, the amount of charge transfer between electrodes EG2 and EG1 can be expressed as follows:

(7) $$Q=\int_{0}^{l}\frac{\sigma bds}{1+{\left(\frac{C_{2}\left(s\right)}{C_{1}\left(s\right)}\right)}_{x=x(t)}}-\int_{0}^{l}\frac{\sigma bds}{1+{\left(\frac{C_{2}\left(s\right)}{C_{1}\left(s\right)}\right)}_{x=0}}.$$

When the sliding distance is x(t), capacitors $C_{1}\left(s\right)$ and $C_{2}\left(s\right)$ can be expressed as:

(8) $$C_{1}\left(s\right)=\left\{\begin{array}{c} \frac{\varepsilon \varepsilon_{0}b\left(l-x\left(t\right)\right)}{h},0\leq x\left(t\right)\leq l\\ 0,l\leq x(t)\leq g+l \end{array}\right.$$

(9) $$C_{2}\left(s\right)=\left\{\begin{array}{cc} 0, & 0\leq x(t)\leq g\\ \frac{\varepsilon \varepsilon_{0}b\left(x\left(t\right)-g\right)}{h}, & g<x(t)\leq g+l, \end{array}\right.$$

where ε is the dielectric constant of the triboelectric layer film, $\varepsilon_{0}$ is the dielectric constant of the vacuum environment, *g* is the distance between the adjacent grid-like electrodes EG1 and EG2, and *h* is the distance of the gap between the triboelectric PTFE layer and the electrode. By combining Formulas (7)–(9), the expression for the charge transferred between the two electrodes can be obtained as follows:

(10) $$Q=\left\{\begin{array}{c} -\sigma bl,\;0\leq x(t)<g\\ \frac{x(t)-l}{l-g}\sigma bl,\;g\leq x\left(t\right)<l\\ \sigma bl,\;l\leq x(t)<g+l. \end{array}\right.$$

When the distance *g* between the adjacent grid-like electrodes EG1 and EG2 is much smaller than the electrode width *l*, Formula (10) can be simplified as follows:

(11) $$Q=\frac{x(t)-l}{l}\sigma bl=\left[x\left(t\right)-l\right]\sigma b.$$

According to Formula (11), the charge is linearly related to the displacement, meaning it is associated with the position of the triboelectric layer. Since the current is the amount of charge passing through the conductor’s cross-section per unit time, the following relationship can be derived:

(12) $$\begin{array}{c} I=\frac{dQ}{dt}=\frac{d(x(t))}{dt}\sigma b=\sigma bv \end{array},$$

where *t* is time and *v* is the relative motion speed between the triboelectric PTFE layer and the metal copper electrode. It can be observed that, in the case of a closed-loop circuit, the faster the relative speed between the triboelectric layer and the metal electrode is, the greater the current is. The voltage across the external load can be expressed as follows:

(13) $$U=IR_{load}=\sigma b\nu R_{load},$$

where $R_{l}oad$ is the load resistance of the self-sensing device.

The sensitivity of the sensor can be expressed as follows:

(14) $$S=\frac{dU}{d\nu }=\frac{d\left(\sigma b\nu R_{load}\right)}{d\nu }=\sigma bR_{load}.$$

By deriving the above formulas, it can be concluded that, in the case of a sensing device connected to a load, the amount of the charge transfer is related to the relative movement position. The amount of charge moved per unit time correlates with velocity; thus, the load voltage in the external circuit can reflect the magnitude of the relative motion speed.

### 2.3. The Relationship between Voltage and Displacement

In an open-circuit scenario, we analyze the relationship between the voltage output characteristics of the sensing device and the motion state. Due to the insulating nature of PTFE, charges are uniformly distributed on its surface, and their decay over time can be neglected. In the design developed in this study, the gap between the electrode plates is much smaller than the width of the plates, which can be ignored when analyzing the relative motion between the triboelectric layer and the electrode. If the surface charge density of the triboelectric layer is −σ, the sum of the charges on the contact surface of the triboelectric layer and the metal electrode overlapping the surface can be regarded as 0. Since it is an open circuit, and the metal electrode maintains charge conservation, the amount of charge on the non-overlapping surface of metal electrode EG1 is −σ(l−x). Similarly, the amount of charge on the non-overlapping surface of metal electrode EG2 is −σx. Thus, the charge density on the non-overlapping surface of electrode EG1 is as follows:

(15) $$\rho_{1}=-\sigma \frac{l-x}{x}.$$

The charge density on the overlapping surface of electrode EG1 is as follows:

(16) $$\rho_{2}=\sigma .$$

The charge density on the overlapping surface of electrode EG2 is as follows:

(17) $$\rho_{3}=\sigma .$$

The charge density on the non-overlapped surface of electrode EG2 is as follows:

(18) $$\rho_{4}=-\sigma \frac{x}{l-x}.$$

Using the above charge distribution law and Gauss theorem, the open-circuit voltage between electrodes EG1 and EG2 can be obtained as follows:

(19) $$V_{\alpha }=U_{EG1}-U_{EG2}=\sigma \left(\frac{x}{l-x}-\frac{l-x}{x}\right)\frac{d_{0}}{\varepsilon_{0}},$$

where $d_{0}$ is the effective thickness constant [33], which is the sum of the ratio of the thickness of the dielectric material to its relative permittivity, and $\varepsilon_{0}$ is the permittivity of the vacuum.

**Figure 4:**
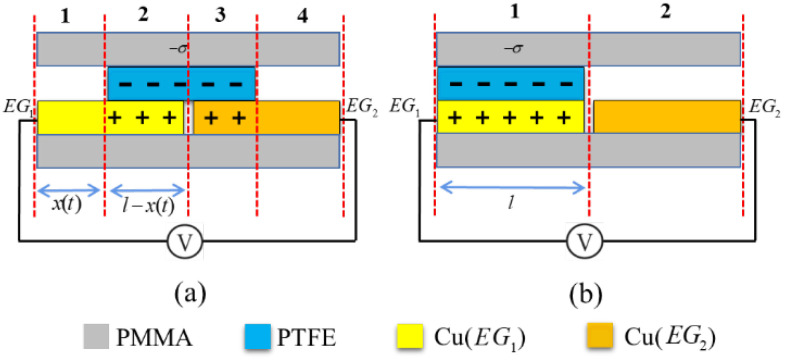
Charge transfer of a TENG in an open circuit. (**a**) non-overlapping state, (**b**) overlapping state.

In the above derivation, when the triboelectric layer tends to overlap with the electrode, the denominator in the formula tends toward 0, evidently making it inapplicable. This is because the total charge on the electrode remains conserved, leading to a substantial accumulation of charges in an extremely small, non-overlapping region. Therefore, Formula (19) is only used to illustrate the trend of $V_{oc}$ during the independent layer TENG motion [34]. A case where the metal electrode overlaps with the triboelectric layer is discussed separately. As shown in Figure 4b, the surface charge density of the triboelectric layer is −σ, resulting in a charge density of σ on the metal electrode surface. Since both the triboelectric layer and the electrode are thin, the total charge on the contact surface of the two is 0. According to the principles of charge conservation within conductors and electrostatic induction, electrode EG1 can be treated as an equipotential body with a charge density of −σ. In the previous rotation process, this portion of the charge is uniformly distributed in the non-overlapping region. Thus, the potentials of electrodes EG1 and EG2 with respect to the reference point at infinity can be calculated, respectively, using the following two formulas:

(20) $$U_{1}=-\sigma \frac{d_{0}}{\varepsilon_{0}}$$

(21) $$U_{2}=0.$$

Thus, when the triboelectric layer coincides with electrode EG1, the open-circuit voltage between the two electrodes is as follows:

(22) $$V_{oc}=U_{1}-U_{2}=-\sigma \frac{d_{0}}{\varepsilon_{0}}.$$

According to Formula (19), it can be observed that the open-circuit voltage $V_{oc}$ of the sensing device is positively correlated with the motion displacement *x*. $V_{oc}$ increases with the increase in *x*, which can also be demonstrated by derivation of the formula. The maximum open-circuit voltage $V_{ocmax}$ is located at the position where the triboelectric layer coincides with the electrode. According to Formula (19), $V_{ocmax}$ is related to the charge density σ, effective thickness constant $d_{0}$, and the permittivity of the vacuum $\varepsilon_{0}$, which are all inherent parameters of the TENG. Therefore, the maximum open-circuit voltage of the TENG is a constant value, independent of velocity and displacement.

## 3. Simulation

For a more comprehensive analysis of the sensing characteristics of the sensing device and an investigation into both the variations in potential and the transfer of charges during its operation, finite-element simulations of a grid-like TENG with a freestanding triboelectric layer mode in an expanded state were performed using COMSOL.

**Figure 5:**
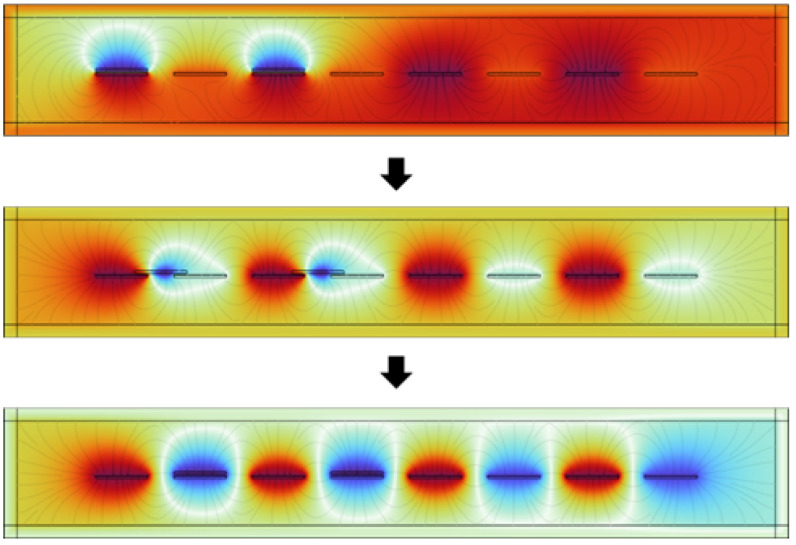
Simulation of a TENG in working state. Red represents a positive electric potential, while blue represents a negative electric potential.

As shown in Figure 5, a model is constructed in a two-dimensional plane. The grid-like triboelectric layer is located in the upper part, consisting of two grid bar triboelectric units that are interconnected. The material of the grid bar is PTFE, and the surface charge density is set to $-1\times 10^{-7}\;C$. The thickness of a single grid bar triboelectric unit is 1 mm, with a length and width of 20 mm. The distance between the two triboelectric layers is 40 mm. In the lower part, there are two interlaced grid electrodes made of copper. Each grid electrode consists of four grid bar units, and the four grid bars of the same grid electrode are also interconnected. The thickness of a single grid bar is 1 mm, with a length and width of 20 mm. The physical field is selected as the electrostatic field. Since the variables contain time-dependent quantities, the study type is set to transient analysis. The space-filling material is air, and the relative permittivity is 1. Due to triboelectrification and charge conservation, the total charge carried by the grid electrodes is the same as the total charge in the triboelectric layer, but the polarity of the electrodes is opposite. The electrode initially coinciding with the triboelectric layer is labeled as EG1, with a charge of $1\times 10^{-9}\;C$.

### 3.1. The Relationship between Voltage and Displacement and Velocity

**Figure 6:**
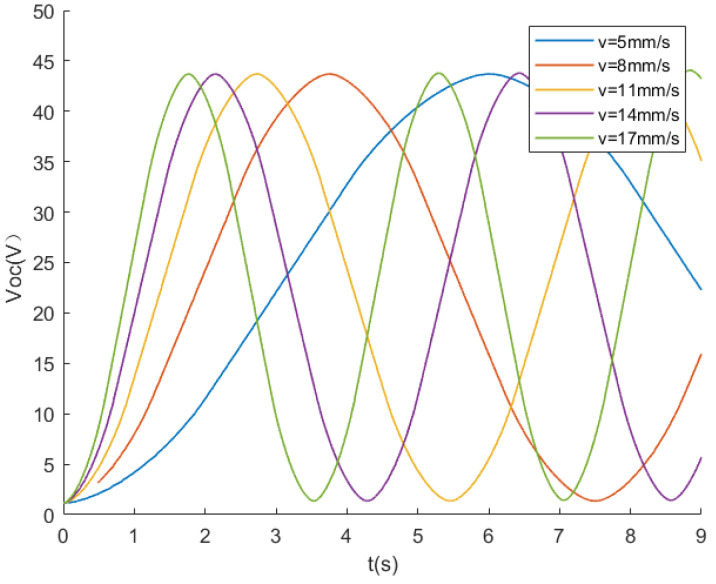
Effect of speed on open-circuit voltage.

After setting up the triboelectric layer and electrodes, an analysis of the open-circuit voltage output characteristics of the sensing device is conducted. The lower surface of the two electrodes EG1 and EG2 is set as the suspension potential, and the potential difference between the two electrodes is the open-circuit voltage $V_{oc}$ of the TENG. The triboelectric layer is slid, relative to the electrode, at five different speeds: 5, 8, 11, 14, and 17 mm/s. Figure 6 shows the numerical values of the open-circuit voltage $V_{oc}$ at different speeds.

From the graph, it can be observed that, at a constant speed, the voltage exhibits periodic increases or decreases as the triboelectric layer moves. As the speed increases, the slope of the open-circuit voltage curve continuously rises, indicating that the voltage increment $▵V_{oc}$ increases with speed. However, the maximum value of $V_{oc}$ remains constant regardless of the speed increase. This is because the potential difference between the electrodes is related to the charge on the electrode plates, and the relative speed between the triboelectric layer and the electrodes does not alter the total charge on the electrode plates. Thus, the maximum potential difference between the two plates always occurs in the region where the triboelectric layer and the electrodes overlap.

Through Figure 6, it can be observed that, during the process of the triboelectric layer sliding from one electrode grid to the next—which corresponds to one step—the open-circuit voltage $V_{oc}$ of the TENG is only related to the position of the triboelectric layer and is independent of the relative motion speed.

### 3.2. The Relationship between Charge Transfer, Displacement, and Velocity

In the previous section, the relationship between the open-circuit voltage $V_{oc}$ and the motion state was analyzed. The relationship between the motion state and transferred charge is analyzed in this section. In the simulation, it is necessary to ground the lower surfaces of both electrodes EG1 and EG2. These grounded plates represent a short circuit between the electrodes. The amount of charge flowing into the ground terminal from one electrode is equal to the amount of charge flowing into the other electrode. Therefore, the amount of charge entering (or leaving) one plate is equal to the amount of charge transferred between the plates.

**Figure 7:**
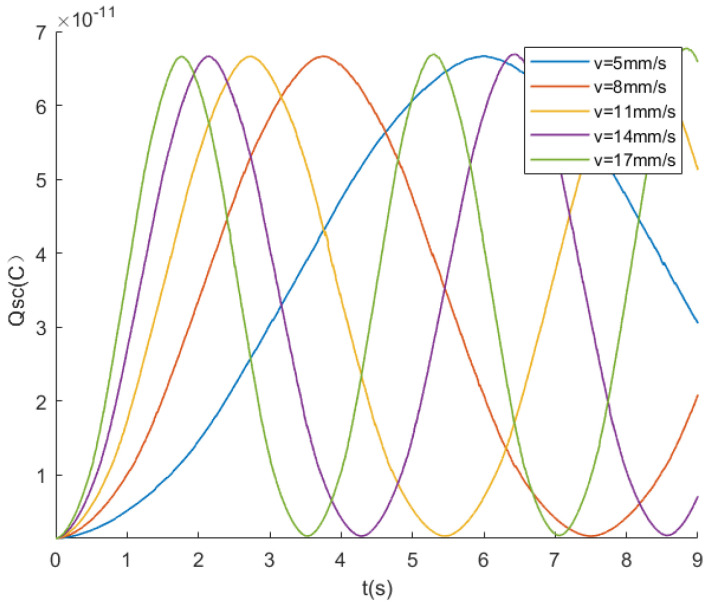
Charge transfer at different speeds.

The triboelectric layer slides relative to the electrode layer at speeds of 5, 8, 11, 14, and 17 mm/s, as shown in Figure 7. The plot illustrates the charge transfer amount $Q_{sc}$ between two electrodes at different speeds. The trend of the charge transfer is similar to that of the open-circuit voltage $V_{oc}$ in Figure 6. When the triboelectric layer moves at a constant speed relative to the electrode layer, the voltage increases or decreases periodically. As the speed increases, the slope of the charge transfer curve grows continuously, indicating that the increment $▵Q_{sc}$ increases with velocity. However, the maximum value of $Q_{sc}$ at different speeds remains constant. This is because the charge generated by friction is fixed, and motion only results in the transfer of charges between electrodes, without the generation of new ones. Similar to the relationship between open-circuit voltage and displacement, the charge transfer quantity $Q_{sc}$ is position dependent within a step, independent of speed.

**Figure 8:**
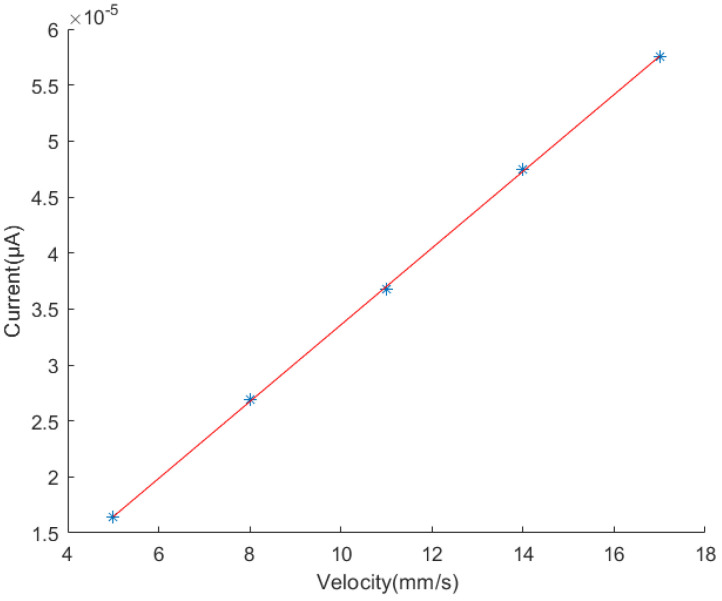
The relationship between current and velocity.

In a circuit, the current represents the amount of charge passing through the conductor’s cross-sectional area per second. The charge amount $Q_{sc}$ on the electrode is an accumulation value. The incremental $▵Q_{sc}$ is an instantaneous value. The instantaneous current value between the two electrodes per unit time, represented by the increment $▵Q_{sc}$, can be approximated by the slope of the charge transfer curve in Figure 7. It can be observed from Figure 7 that the intervals of charge growth or decline tend to be approximately linear, as previously derived in the formulas. The curvature at the endpoints is caused by the gap between the electrode plates, an effect that is analyzed later in this paper. Selecting the midpoint of each step and calculating the slope can be carried out to ascertain the amount of charge passing through per unit time. By calculating the charge increment per unit time at different velocities, the relationship between current values and velocity can be obtained, as shown in Figure 8. It is evident from the graph that there is a linear correlation between the motion speed and the current output, consistent with the earlier-derived conclusions.

The slopes of the lines at different velocities in Figure 7 represent the different current values. In the previous discussion, the linear relationship between current and velocity is based on the condition that the gap *g* between the electrodes is much smaller than the width *l* of the electrodes. To verify the impact of the gap *g* on the charge transfer, simulations are conducted using COMSOL.

**Figure 9:**
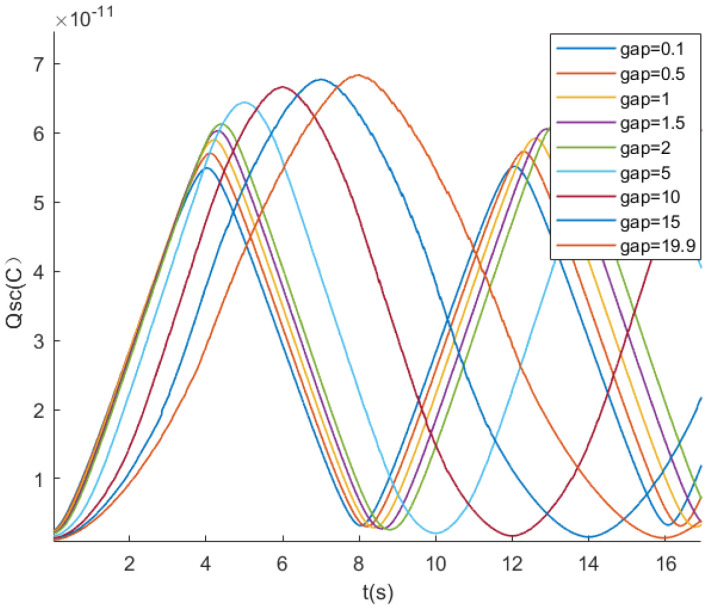
Effect of electrode spacing on charge transfer.

The electrodes slide relative to the triboelectric layer at a velocity of 5 mm/s. The gaps *g* between the two electrodes are set to 0.1, 0.5, 1, 1.5, 2, 5, 10, 15, and 19.9 mm, while the other parameters are kept constant. As shown in Figure 9, with the increase in the gap, the angle at the inflection point of the curve tends to be smooth, and linearity deteriorates. In addition, it can also be found that the peak value of $Q_{sc}$ also increases. In the interval of 0.1 to 2, the linearity does not change much, and the peak value of $Q_{sc}$ increases by $6\times 10^{-12}\;C$. In the interval of 2 to 19.9, the peak value of $Q_{sc}$ only increases by $7.5\times 10^{-12}\;C$, and the linearity change is more obvious. In conclusion, when the gap is small, the change of gap has an obvious influence on the peak value of $Q_{sc}$. When the gap is large, the change of the gap has an obvious influence on the linearity.

According to the simulations, the amount of charge transfer is position dependent and linearly related to displacement within one step, while the current value is linearly related to velocity. Since the output voltage is a product of the load and current, it can be concluded that the output voltage is linearly related to velocity.

## 4. Experiments and Analysis

### 4.1. Frequency Characteristic Experiment of Sensing Device

Through the theoretical derivation and simulation presented earlier, it can be concluded that the maximum open-circuit voltage of the sensing device is a constant value, making it a suitable representation of the frequency measurement. The following experimental work focused on testing the frequency characteristics of the sensing device.

**Figure 10:**
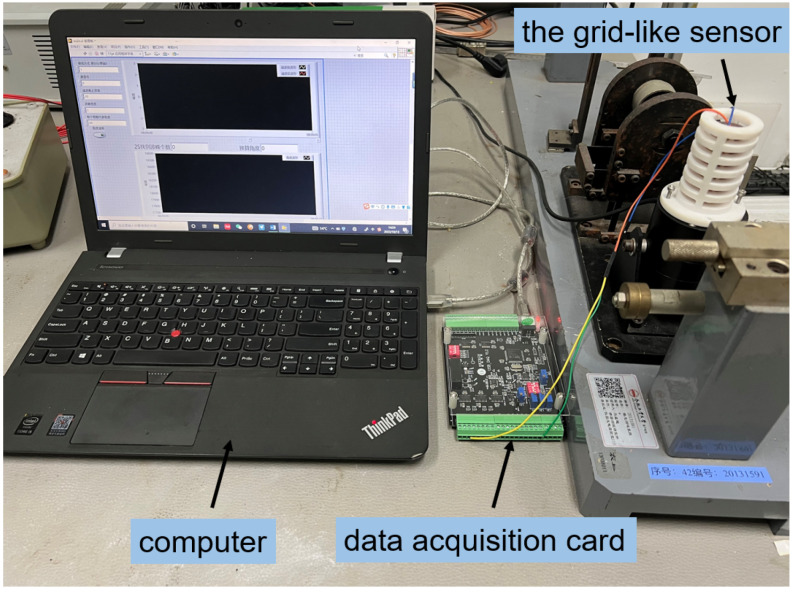
Frequency monitoring test of the sensing device.

The triboelectric layer in the sensing device is fabricated from PTFE material, with each individual grating strip having a width of 5 mm. The metal electrodes are made by attaching copper foil to an acrylic cylindrical sleeve, with each individual grating strip having a width of 4 mm. The sensing device is mounted on a small exciter, with the triboelectric layer attached to the exciter’s casing, and the sleeve with electrodes connected to the exciter’s top rod. As shown in Figure 10, the two pins of the copper electrodes are connected by leads to a data acquisition card for signal collection and transmission to a host computer. The signals are then analyzed using LABVIEW 2018 to obtain information such as amplitude and frequency.

**Figure 11:**
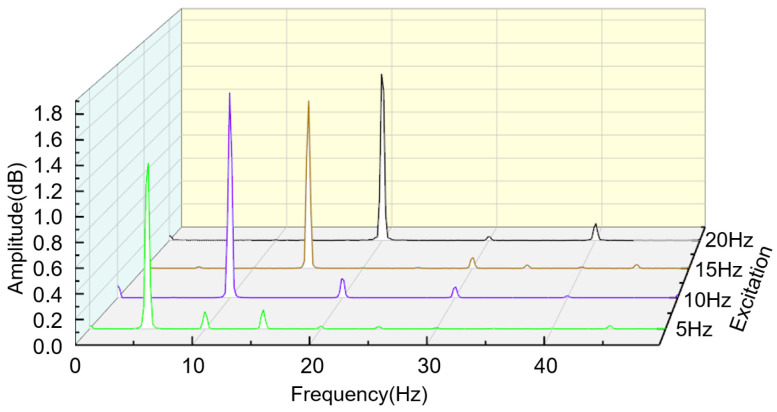
Amplitude–frequency curve corresponding to the output electrical signal.

**Figure 12:**
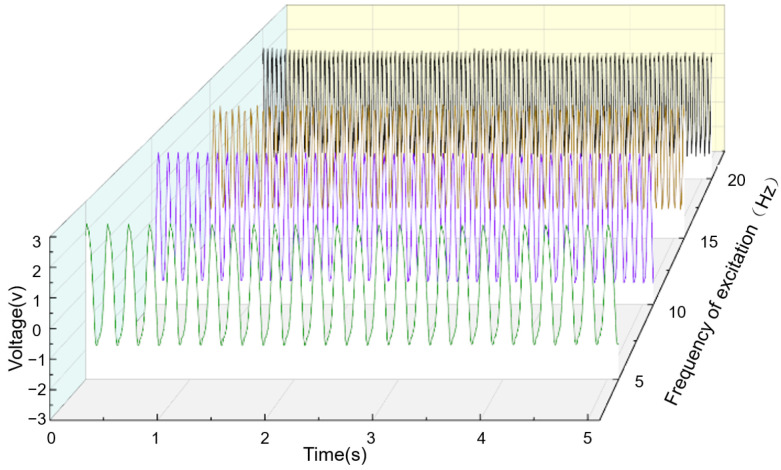
Output voltage of the sensing device under different frequency excitations.

In the experiment, the frequency and power output of the exciter are adjusted to maintain an amplitude of 5 mm at frequencies of 5 Hz, 10 Hz, 15 Hz, and 20 Hz. The open-circuit voltage of the sensing device is also collected. As shown in Figure 10, the open-circuit voltage at the same frequency exhibits periodic changes in the time domain. With an increase in the exciter vibration frequency, the lines representing the output voltage of the sensing device in the graph become more densely packed. Frequency domain analysis of the output voltage signal is shown in Figure 11. It is evident that the frequency of the output signal is concentrated at the vibration frequency, indicating that the sensing device can reflect the vibrational state of the measured object. Additionally, from Figure 12, it can also be observed that the maximum open-circuit voltage output at different excitation frequencies remains essentially unchanged at around 2 V. This validates the earlier derivation that the open-circuit voltage is independent of factors such as frequency and velocity, instead being related to intrinsic properties.

Because the peak value of the open-circuit voltage of the sensing device remains constant with external factors such as frequency and velocity, monitoring the vibration frequency can be achieved by identifying the number of peaks in the unit time through the computer.

The frequency monitoring capability of the sensor device is tested through experiments, which can be achieved through either amplitude–frequency curve monitoring or by recording the number of peaks in the signal. Additionally, the experiment validates the conclusion from the theoretical derivation that the open-circuit voltage is only related to intrinsic properties such as vibration position and material, and is independent of speed, frequency, and other external factors.

### 4.2. Velocity Characteristic Experiment of Sensing Device

**Figure 13:**
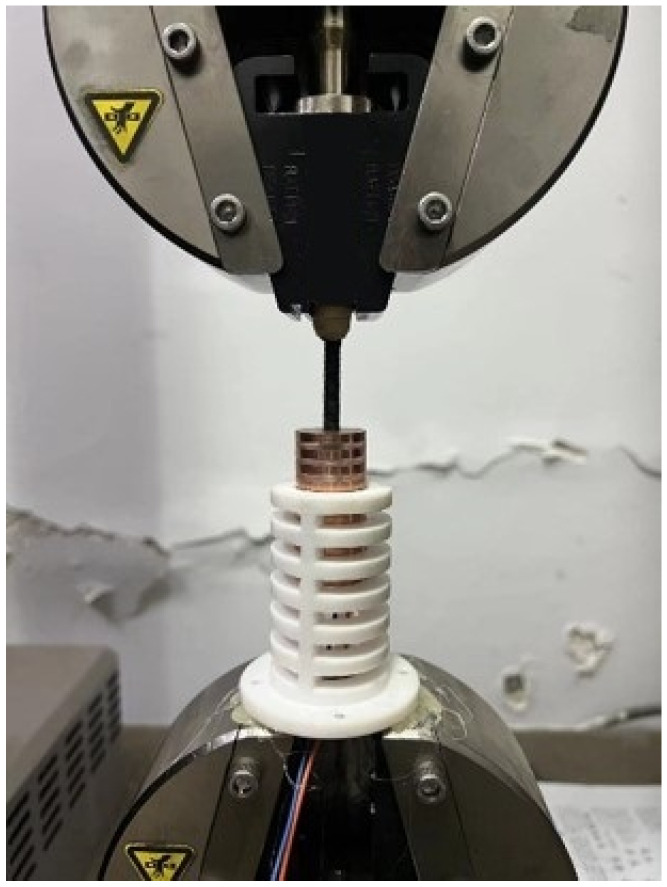
Velocity correlation experiment of sensing device.

In order to validate the speed monitoring capability of the sensing device, it was mounted on an MTS tensile testing machine, as illustrated in Figure 13. The machine was operated at four different speeds, 5, 8, 11, and 14 mm/s, consistent with the simulation settings. As derived in the previous analysis, the velocity is related to the amount of charge transfer per unit time, that is, the current. Due to the characteristics of the TENG, which result in small current values, the voltage across the load resistor was measured.

**Figure 14:**
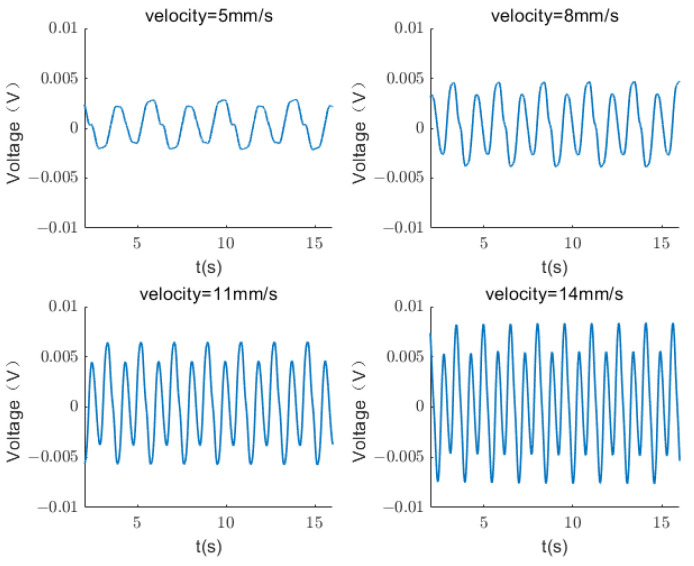
Load voltage of a TENG at different speeds.

Figure 14 displays the load voltage of the sensing device at four different speeds. Under a constant speed, the output signal presents periodicity. As the speed of the testing machine increases, the period shortens, but the amplitude of the load voltage increases. Simultaneously, the number of grids passed per unit time increases; that is, the distance increases, thereby indicating an increase in speed.

**Figure 15:**
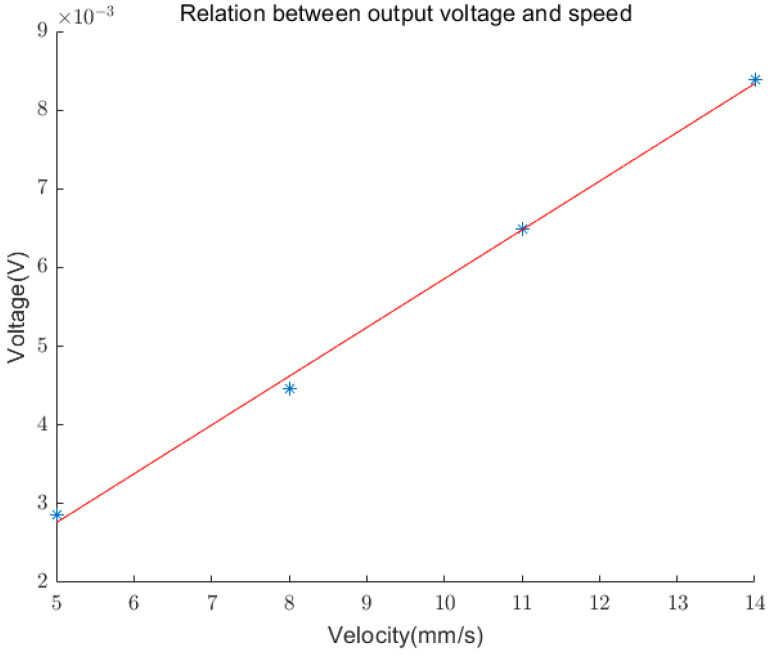
The relationship between the maximum output voltage and speed.

The amplitude of the load voltage at different speeds is represented in Figure 15 and fitted. It can be observed that the maximum load voltage of the sensing device exhibits a linear relationship with the speed of the testing machine.

Through the above experiments, it is evident that the load voltage of the sensing device is linearly related to the velocity of the measured object. For uniform motion, the velocity can be obtained by analyzing the signal’s period per unit time and the width of the grating. For non-uniform motion, the relationship between the amplitude and speed provides a means through which to determine the velocity.

### 4.3. Output Stability Experiment of Sensing Device

The designed sensing device utilizes the periodicity of the TENG’s output signal and the amplitude–velocity correlation to measure frequency and speed. Signal attenuation and instability can impact the accuracy of measurements. As the triboelectric layer acts as a polarized material, the surface charge stabilizes after reaching saturation during contact electrification. Additionally, the electrostatic induction effect only guides the transfer of charges between the electrodes, without generating or consuming charges, thereby ensuring the stability of the sensing device’s output.

The designed sensor takes advantage of the periodicity and amplitude–velocity correlation of the output electrical signal of the grid-like TENG to measure the frequency and speed, and the signal output attenuation and instability affect the measurement accuracy. Because the triboelectric layer is an electret, the charge on its surface tends to be stable and can be maintained for a long time after reaching saturation during contact electrification. Moreover, the triboelectric layer only guides the charge transfer between the electrodes, and there is no charge inflow or outflow itself; thus, in theory, it can ensure the stability of the output of the sensing device.

**Figure 16:**
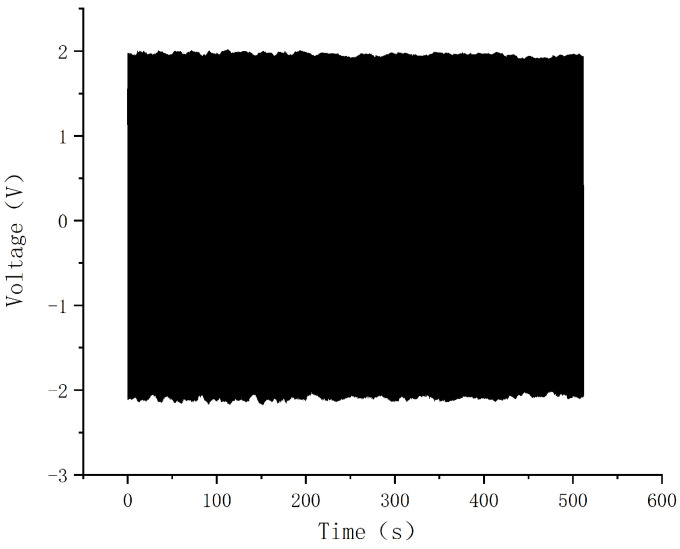
Voltage output for long time vibration under 5 Hz excitation.

To test the output stability of the sensing device, experiments are conducted with an excitation frequency of 5 Hz, an amplitude of 5 mm, and a duration of 512 s. Figure 16 illustrates the open-circuit voltage values of the sensing device, revealing minimal amplitude variations. This observation suggests good repeatability and stability during continuous measurements.

## 5. Conclusions

This paper applied triboelectric nanogenerator (TENG) technology to a sensing device and analyzed the sensing characteristics of a grid-shaped TENG in freestanding triboelectric layer working mode. By establishing a model for formula derivation, it is determined that the maximum open-circuit voltage and charge transfer of the sensing device are constant, solely dependent on the inherent parameters of the sensing device. The amount of charge transfer per unit time—that is, the current—exhibits a linear relationship with the motion velocity. Subsequently, the working state of the sensing device was simulated using COMSOL finite-element simulation software. The obtained results are consistent with the theoretical derivations. The simulation also analyzed the impact of the gaps between electrodes on the linearity of the amount of charge transfer, providing a basis for improving sensing accuracy in sensor design. Finally, performance tests for frequency and velocity monitoring were conducted on the exciter and testing machine. The experimental results indicate that the voltage output signal of the sensing device exhibits periodicity with displacement. The maximum open-circuit voltage output is independent of the velocity and frequency and only correlates with the inherent properties. Additionally, the maximum load voltage output demonstrates a linear relationship with the velocity.

Through theoretical analysis and simulation experiments, it can be concluded that grid-like sensors based on the TENG can be used to monitor motion frequency, displacement, and velocity. In practical applications, these sensors can be integrated into a vehicle’s suspension for the detection of the vehicle’s vibration state or integrated on the columns of offshore platforms for the detection of wave undulation. Because the triboelectric and electrode layers can be made very thin, there is no need for major changes to be made to the target structure, thereby reducing the complexity of these sensors’ integration in the above scenarios. At the same time, because these sensors do not require an energy supply to output signals, they can realize wireless distributed sensing and, therefore, have good application prospects.

## Figures and Tables

**Table 1:** Parameters of the angular velocity of the self-sensing structure.

Structural Parameters	Dimensions (mm)
Axial length of electrode, a	54
Length of single-electrode grid, b	60
Axial length of triboelectric layer, c	65
Length of triboelectric layer, d	75.4
Width of triboelectric layer grid, e	5
Vertical gap in triboelectric layer, f	5
Horizontal gap in triboelectric layer, g	32.7
Width of single-electrode grid, h	3
Horizontal gap between adjacent electrode grids, i	1
Vertical gap between adjacent electrode grids, j	2

## Data Availability

The data presented in this study are available on request from the corresponding author.
